# Artificial Intelligence/Machine Learning-Driven Small Molecule Repurposing via Off-Target Prediction and Transcriptomics

**DOI:** 10.3390/toxics11100875

**Published:** 2023-10-22

**Authors:** Mohan Rao, Eric McDuffie, Clifford Sachs

**Affiliations:** Neurocrine Biosciences, Inc., Nonclinical Toxicology, San Diego, CA 92130, USA; emcduffie@neurocrine.com (E.M.); csachs@neurocrine.com (C.S.)

**Keywords:** drug repurposing, off-target prediction, AI/ML, cheminformatics, transcriptomics, due diligence, drug life cycle management

## Abstract

The process of discovering small molecule drugs involves screening numerous compounds and optimizing the most promising ones, both in vitro and in vivo. However, approximately 90% of these optimized candidates fail during trials due to unexpected toxicity or insufficient efficacy. Current concepts with respect to drug–protein interactions suggest that each small molecule interacts with an average of 6–11 targets. This implies that approved drugs and even discontinued compounds could be repurposed by leveraging their interactions with unintended targets. Therefore, we developed a computational repurposing framework for small molecules, which combines artificial intelligence/machine learning (AI/ML)-based and chemical similarity-based target prediction methods with cross-species transcriptomics information. This repurposing methodology incorporates eight distinct target prediction methods, including three machine learning methods. By using multiple orthogonal methods for a “dataset” composed of 2766 FDA-approved drugs targeting multiple therapeutic target classes, we identified 27,371 off-target interactions involving 2013 protein targets (i.e., an average of around 10 interactions per drug). Relative to the drugs in the dataset, we identified 150,620 structurally similar compounds. The highest number of predicted interactions were for drugs targeting G protein-coupled receptors (GPCRs), enzymes, and kinases with 10,648, 4081, and 3678 interactions, respectively. Notably, 17,283 (63%) of the off-target interactions have been confirmed in vitro. Approximately 4000 interactions had an IC_50_ of <100 nM for 1105 FDA-approved drugs and 1661 interactions had an IC_50_ of <10 nM for 696 FDA-approved drugs. Together, the confirmation of numerous predicted interactions and the exploration of tissue-specific expression patterns in human and animal tissues offer insights into potential drug repurposing for new therapeutic applications.

## 1. Introduction

The process of small molecule drug discovery often begins with screening a large number of compounds, followed by optimizing the most promising candidates, known as lead candidates [1,2,3]. In particular, the past decade has seen significant advancement in drug discovery technologies for the identification of lead candidates that can target various therapeutic modalities to disrupt protein–protein interactions, such as protein degraders and antibody drug conjugates. Despite these advances, recent findings from the Innovation and Quality (IQ) Consortium reveal that a sizable proportion of such lead candidates encounter significant failure in the later stages of discovery. This failure was attributed to unfavorable interactions with unintended proteins or inadequate physicochemical properties [4]. To address these issues, newer tools such as high-throughput chemistry techniques [5], artificial intelligence/machine learning (AI/ML) [6,7], computational docking [8], free energy calculations [9,10], and protein Structure–Activity Relationship (SAR) drug design tools have been utilized to produce compounds that demonstrate target-specific engagement in vitro.

However, despite the use of these tools, many lead candidate compounds may not favorably engage the intended target for efficacy in animal models due to factors such as high molecular weights and flat or high hydrophilicities [11,12]. This necessitates further optimization to observe efficacy in both animal models and humans. Despite significant investments of over USD 2.5 billion (about USD 8 per person in the United States of America (USA)) per approved drug, only a small fraction of compounds that enter Phase I clinical trials are eventually approved for marketing by regulatory agencies such as the USA Food and Drug Administration (FDA) [13,14]. Furthermore, the recent New Clinical Development Success Rates Report revealed that between 2011 and 2020 of the 12,728 compounds in various therapeutic areas, the average success rate from Phase I to FDA approval was only 7.9%. Other success rates included 15.5% for metabolic compounds; between 13.2% and 8.3% for infectious disease, ophthalmology, autoimmune, allergy, and gastroenterology compounds; 7.5% for respiratory compounds; and 7.3% for psychiatry compounds. Significant compound attrition was evident for endocrine, neurology, oncology, cardiovascular, and urology compounds, indicating a success rate that fell below 6%. The top contributing factors toward success were disease indication, target, modality, and drug novelty.

Analysis of 16 major research-based pharmaceutical companies [15] revealed increased annual R&D spending at 6%, with an average expenditure of USD 6.7 billion per company in 2020. However, despite this substantial investment, these companies only managed to launch a total of 251 new drugs over the 20-year period (2001–2020), averaging around 12 drugs per year. Therefore, nonclinical testing continues to reveal challenges for drug hunters in the pharmaceutical industry, with high failure rates due to safety concerns and/or poor efficacy in humans [16]. Because of significant compound attrition during the discovery phase, more pharmaceutical companies have turned to a process known as drug repurposing or drug repositioning. This involves identifying unintended target(s) interactions within specific tissues of interest (e.g., the central nervous system (CNS)) or intended targets expressed in “off-tissues” (i.e., other than the original tissues a compound was first designed to evoke protein interaction(s) in), with the goal of discovering novel applications for existing FDA-approved drugs [17,18,19]. This approach is generally faster and more cost-effective than traditional drug research and development processes. For instance, a CNS drug that has already been approved for one indication, once demonstrated to be efficacious for unrelated disease, may be repurposed for a different indication such as endocrinology [20]. Additionally, drug repurposing has the potential to address unmet medical needs more rapidly, including rare diseases for which there are currently no effective oral treatments [21].

In recent years, significant advances have occurred in the development of novel AI/ML approaches to enable the analysis of large datasets including in vitro, in vivo, ligand interactions, antibody–antigen interactions, protein structures, biochemical, and baseline gene expression profiles in different tissues (or cells) across various species, to rapidly identify potential new uses for approved drugs [19,22,23]. AI/ML-based approaches are valuable tools to predict potential therapeutically valuable off-target interactions for small molecule drugs [24]. Improving our ability to predict off-target interactions has the potential to increase the success of small molecule drug development by identifying new targets and therapeutic indications in vitro and in vivo. To address this need, we developed a computational framework that combines AI/ML chemical structure-based methods and protein binding site-based target prediction approaches to predict potential off-target interactions and their tissue expression information across species. We applied this novel framework to predict potential repurposing opportunities for 2766 FDA-approved small molecule drugs (i.e., collectively referenced often within this manuscript as “the dataset”).

## 2. Materials and Methods

**Figure 1:**
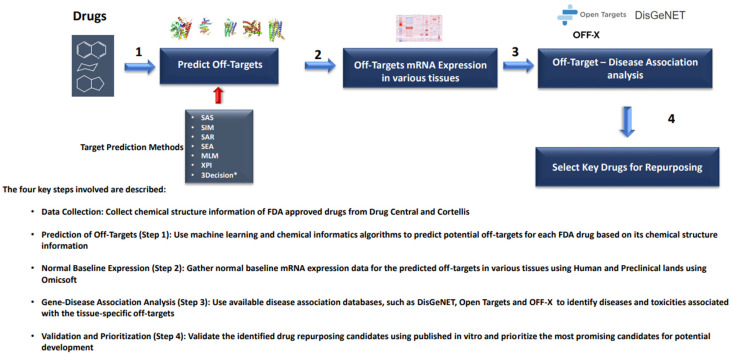
Schematic representation of computational drug repurposing workflow.

The drug repurposing computational framework involves a hierarchical approach, as shown in Figure 1. The first step is two-dimensional (2D) chemical similarity analysis, where potential off-target proteins for 2766 FDA-approved drugs were profiled using various cheminformatics and machine learning methods, including Similarity Active Subgraphs (SAS) [25], SAS-based Quantitative Structure-Activity Relationship (SAR) models [26], Molecular Similarity (SIM) [27], Similarity Ensemble Approach (SEA) [28], Machine Learning (ML) methods, and Cross Pharmacology Indices (XPI) [29]. The machine learning methods [30] included Random Forest (RF), Artificial Neural Network (aNN), and Support Vector Machine (SVM). Collectively, this so-called Off-Target Safety Analysis (OTSA) process predicts off-target binding targets that may have efficacious or unintended adverse effects.

Below, we describe each of the 6 cheminformatic and three-dimensional (3D) methodologies used in the drug repurposing process:

SAS: The SAS methodology involves identifying the simplest active subgraph containing the minimum pharmacophoric features required to achieve activity for a given biological target. This approach facilitates the identification of similar pairs of molecules, which were previously categorized as dissimilar, thereby expanding their applicability domain and improving prediction. Also, the SAS methodology helps prevent the identification of similar artifacts and reduces the impact of false positives, thereby improving precision [25].

SAR: The SAR methodology facilitates the development of large-scale QSAR (Quantitative Structure–Activity Relationship) models for each target family. This approach is particularly useful for kinases, GPCRs, ion channels, proteases, transporters, immunoglobulin receptors, and other target families.

SIM: The SIM methodology utilizes three types of 2D descriptors to compute the similarity between two chemical structures. These descriptors include Pharmacophoric Fragments (PHRAG), Feature-Pair Distributions (FPD), and Shannon Entropy Descriptors (SHED). Each descriptor characterizes chemical structures with a different degree of randomness that complements the others in terms of chemical structural similarity [26].

SEA: The SEA methodology identifies related proteins based on set-wise chemical similarity among the ligands. It has been shown to be effective in predicting novel ligand–target interactions using chemical structure information alone [28].

MLM: The MLM methodology leverages more than a thousand high-quality machine learning classifiers that were generated based on FPD molecular descriptors for qualitative binding prediction. The MLM approach is a consensus score of three machine learning models (aNN, SVM, and RF). If the consensus score results are positive, the corresponding ligand–target link is considered likely [30].

XPI: The XPI methodology uses cross-pharmacological data for thousands of small molecules on many different biological targets to enable an in-depth cross-pharmacology analysis [29].

We used the Tanimoto similarity distance to compare the SAS fingerprints of a specific compound with the precomputed archived SAS fingerprints in our reference database. This similarity metric was also employed in SAR, SIM, XPI, and SEA analyses when identifying similar compounds in the reference database.

The computational process for predicting targets involves a two-step approach that utilizes both 2D chemical similarity methods and 3D protein structure-based approaches [16,31]. Published protein structures are accessed through a web-based application known as 3Decision, developed by Discngine S.A.S (Paris, France). The predicted off-targets are ranked based on a normalized pseudo-score that considers the scores from different ligand-centric methods. A pseudo-score of 1.00 indicates high certainty and is assigned when the test molecule is present in the training set with the same mechanism of action or if most of the methods predict a specific target as a likely binding partner. For the present off-target analysis, a pseudo-score of ≥0.55 is considered significant. The 3Decision approach is not included in this work as score normalization because it uses geometric and energy terms to assess each protein–ligand complex, including geometrical features such as shape, size, binding site length, width, depth, hydrophobic patches, nature of amino acid residues, number of hydrogen bond donors and acceptors, and interaction energy. The 3Decision results help to confirm the results from the 2D methodologies, and target prediction was performed for only 14 selected drugs because 3Decision is not a high-throughput process. The results from this approach are then compared to those from the 2D methods. We further assessed the predictive capability of the OTSA process by examining its ability to identify both intended and unintended pharmacological targets. In addition, besides assessing the primary targets, we also examined its predictive power for secondary (i.e., off-target protein) hits. We compared the predicted secondary off-targets to the in vitro binding and functional activity data that were available in the literature.

The drug repurposing process involves using normal human tissue transcriptomic profiles to validate predicted off-target proteins [17,24,32]. This is achieved by combining gene expression profiles of various normal human tissues with the target prediction workflow, which allows for the refinement of the prediction by eliminating irrelevant predicted off-targets. This process helps to develop more robust hypotheses about potential organ-specific repositioning. To determine if potential unintended off-targets need further investigation in the context of repurposing, three criteria are considered: (1) robust clinical and nonclinical evidence linking the potential off-target protein(s) to a therapeutic outcome, (2) the presence of the off-target(s) in normal human and nonclinical species’ tissues of relevance, and (3) a computed composite score >0.55 from target prediction methods. If these criteria are met, further exploration through in vitro and/or in vivo data interpretation may be necessary.

We used the Drug Central database [33] to obtain FDA-approved drugs. The 3D structures of these drugs were generated using the ligprep module in the Maestro tool (Schrodinger LLC, New York, NY, USA), and their physicochemical properties were analyzed using Qikprop and visualized using TIBCO Spotfire (TIBCO Inc., Palo Alto, CA, USA). Properties such as molecular weight, logP, Topological Polar Surface Area (TPSA), number of rotatable bonds, and hydrogen bond acceptors and donors, were evaluated, as they have been associated with a higher degree of unselectivity. The drug repurposing workflow was then applied to these compounds to generate a list of potential off-targets, which is available in Supplementary Table S1. Interestingly, the process of repurposing drugs involves utilizing various computational methods, including Clarity, which incorporates six target prediction techniques, including three machine learning methods, the Transcriptomics body atlas for humans, mice, rats, dogs, and monkeys, Qikprop, 3Decision, Off-X, and DisGeNET [34]. The aim of this combined computational drug repurposing approach is to highlight the efficacy of different computational strategies in predicting comprehensive lists of off-target proteins. This combinatorial approach also aims to assess normal (baseline) target expression at the mRNA level, across various tissues from humans and animal models, along with evidence from the literature linking off-targets to potential drug repositioning.

## 3. Results

### 3.1. Chemical Space Assessment in 2677 FDA-approved Drugs: Implications for Discovery and Repurposing

**Figure 2:**
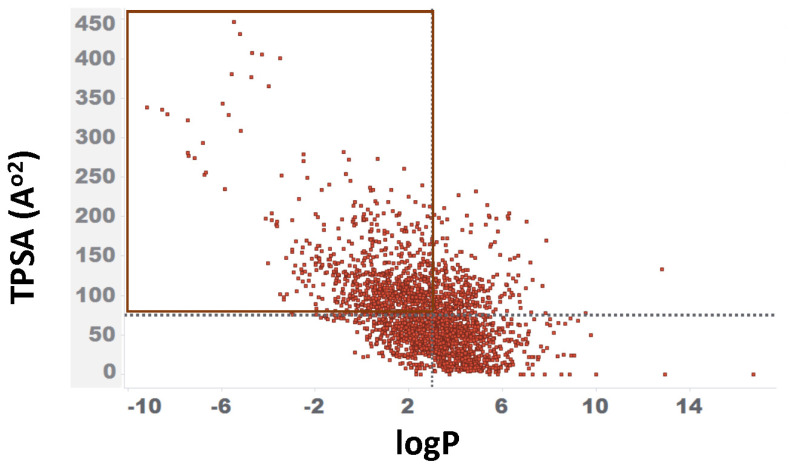
Distribution of logP and TPSA for 2766 drugs. The X-axis displays logP and the Y-axis represents the computed TPSA in A^o2^. The 916 drugs that met the criteria of logP < 3 and TPSA > 75 A^o2^ are highlighted within the red box.

The concept of a “drug-like chemical space” in drug discovery has gained significant attention over the last two decades [35,36,37,38]. Several studies have explored the link between physicochemical property ranges and compound attrition, and their potential impact on technical and regulatory success. For example, drugs with a low logP value (<3) and a high Topological Polar Surface Area (TPSA) (>75 A^o2^) are often less toxic than highly lipophilic compounds, commonly known as the “3/75 rule” [39]. This range is also considered critical for a compound’s ability to cross the blood–brain barrier, a key factor for the successful development of CNS drugs. Consequently, we computed 11 physicochemical properties to determine the extent to which 2766 FDA-approved drugs followed a typical drug-like paradigm. The mean and median values resulting from this analysis are displayed in Table 1. Surprisingly, less than half of these approved drugs followed the “3/75 rule [40].” Figure 2 illustrates the distribution of logP and TPSA values for these drugs. Among them, 37% (916) fully met the criteria of having a logP value of <3 and a TPSA > 75 A^o2^, while 36% (896) exhibited lipophilic characteristics with a logP value of >3 and a TPSA < 75 A^o2^. The remaining 27% had either a logP value of >3 or a TPSA < 75 A^o2^. Taken together, drugs with physicochemical properties outside the typical drug-like range (for example, outside of the 3/75 rule) are still marketed, indicating that physicochemical properties alone did not solely dictate the development of certain drug classes. As a result, in our repurposing analysis, “the dataset” consisted of all 2766 FDA-approved drugs, irrespective of whether they followed to the typical physicochemical properties associated with drug-like compounds.

### 3.2. Predicted Off-Target Interactions in 2766 FDA-approved Drugs: Exploring Potential Repurposing Opportunities

We used the concept of “chemical structural neighbors,” which refers to compounds that share similar structural and pharmacophoric features with a specific drug [24]. By performing chemical similarity and pharmacophoric analysis on approximately 3 million unique reference compounds, we identified 150,620 distinct compounds as chemical structural neighbors to those in the dataset. Our database includes comprehensive information on both on- and off-target protein interactions, including binding and functional data, for the identified structural neighbors. To predict potential off-target interactions, we used a combination of ML and other chemical similarity methods, resulting in 27,371 potential off-target interactions, averaging around 10 interactions for each of the 2766 FDA-approved drugs. It should be noted that although these approved drugs were originally designed for specific protein targets, we revealed an average of 10 interactions, implying the possibility of 9 potential therapeutic or toxicological targets for each drug. Of the 27,371 predicted protein interactions, 17,283 were confirmed through in vitro experiments, representing a confirmation rate of 63%. The 10,088 (37%) predicted off-target interactions have not been verified (or reported by drug developers). For the 63% confirmed (i.e., in vitro) interactions, we summarized the experimentally measured IC_50_ values associated with each interaction in Supplementary Table S1.

Using SAS, SAR, SIM, MLM, XPI, and SEA, resulted in 12,647, 10,804, 15,632, 9918, 13,890, and 8273 protein interactions predicted for the 2766 FDA-approved drugs, respectively. Both the XPI and SEA methods predicted less than 10,000 interactions each, while the other methods predicted more than 10,000 interactions each. Overall, the combined use of these orthogonal methods resulted in 27,361 interactions, with some interactions being predicted by multiple methods and others by only one method. Taken together, these results highlight the importance of using multiple orthogonal methods to effectively capture all potential interactions. Among these interactions, approximately 4000 show high affinity, with a potency of less than 100 nM. The remaining interactions fall within the range of 100 nM to 1 μM. Table 2 provides a comprehensive overview, displaying the target classes together with the respective number of predicted interactions for each class. The most prominent target classes (with the highest number of predicted interactions) were GPCRs, enzymes (proteases, cyclooxygenases, ligases, oxidases, transferases, and others; excluding kinases), and kinases, comprising 38%, 14%, and 13% of total interactions, respectively.

### 3.3. GPCRs

Approximately 56% of non-olfactory-expressed GPCRs have not been evaluated in clinical trials, especially for genetic and immune system disorders. Currently, only about 12% of potentially targetable GPCRs expressed in humans are being targeted by therapeutics in development, with around 100 out of 800 human GPCR targets having at least one marketed drug molecule [41]. This leaves around 700 GPCRs with potential therapeutic, either agonist or antagonist, activity, resulting in a potential total of about 1400 targetable profiles. Therefore, computational off-target prediction processes offer a significant opportunity for discovering new therapeutic agents and repurposing existing ones.

The GPCRs accounted for 38% of all predicted interactions, totaling 10,648 out of the 27,371 predictions. These interactions involved 220 distinct GPCRs and 1471 drugs, which account for 53% of the drug dataset. This translates to an average of 7 interactions per drug. Among the 27,371 predictions, 46% (4942) were confirmed through in vitro experiments, leaving the remaining 54% as unverified high-scoring predictions. Out of a total of 4942 interactions, 2205 of them have affinities of less than 100 nM, which accounts for 46% of all interactions. Among this subset, there are 539 interactions with affinities ranging from 10 nM to 1 nM, and 217 interactions with affinities less than 1 nM. Together, these two categories collectively represent 34% of interactions with affinities less than 100 nM. To validate these affinities, we cross-referenced them with data from the literature. These high-affinity protein interactions have the potential for direct repurposing, while the remaining, much weaker (i.e., μM), affinity interactions can be further optimized for better potency through additional computational structure-based design and synthetic chemistry efforts.

**Figure 3:**
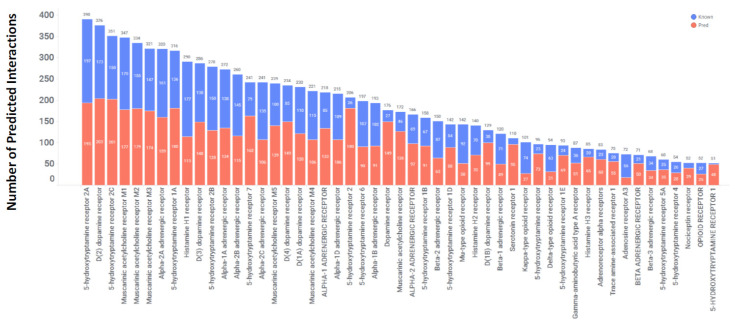
Out of 220 GPCRs, 51 are Predicted with >50 drugs. The X-axis displays gene names and the Y-axis represents the number of drugs interacting with each gene. The blue segment represents the number of predicted interactions that have been in vitro confirmed. The red segment represents high-scoring predicted interactions that have not been in vitro confirmed to date.

Figure 3 shows the top GPCRs, each with more than 50 drug interactions. Interestingly, 51 GPCRs showed interactions with at least 15 different drugs, showing their high non-selective nature and their ability to interact with a chemically diverse drug molecules not originally intended for them. Among the GPCR targets, the muscarinic acetylcholine, serotonin, and dopamine receptors were the most frequently targeted, with the highest number of predicted drug interactions. Approximately 50% of the predicted interactions for each of these targets, as depicted by the blue segment in Figure 3, were experimentally confirmed.

### 3.4. Enzymes

**Figure 4:**
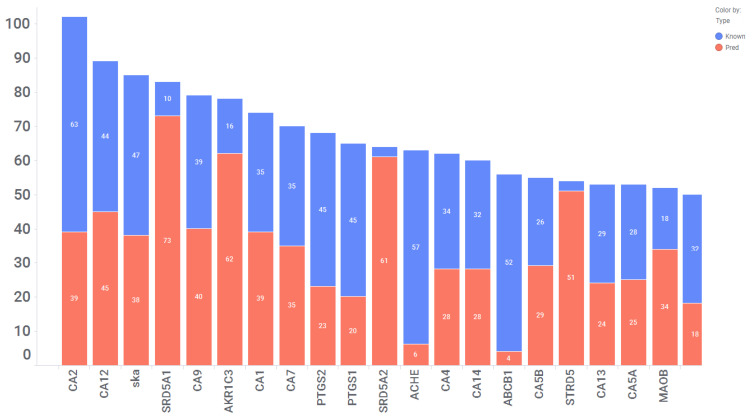
21 Enzymes Predicted to Interact with >50 drugs.The X-axis displays gene names and the Y-axis represents the number of drugs interacting with each gene.The blue segment represents the number of predicted interactions that have been in vitro confirmed. The red segment represents high-scoring predicted interactions that have not been in vitro confirmed to date.

**Figure 5:**
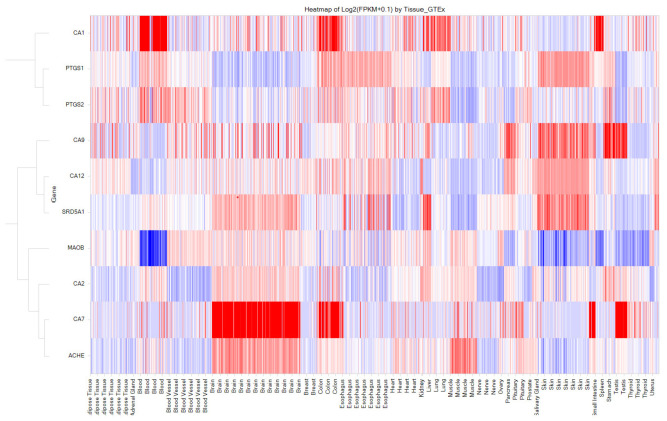
Human Baseline mRNA Expression of Selected 10 Enzymes Interacted with Over 50 Drug Molecules. Among the 10 enzymes, CA7 (Carbonic Anhydrase 7) and AcHE (Acetylcholinesterase) are relatively highly expressed in the brain. The X-axis displays the names of human tissues and the Y-axis represents enzyme names. The Y-axis is hierarchically clustered based on gene expression patterns in various tissues. The color gradient from red to blue indicates high to low expression levels.

Our computational approach included 560 distinct enzymes, including proteases, phosphodiesterases, carbonic anhydrases, cyclooxygenases, acetylcholinesterase, and several classes. These enzymes serve as important therapeutic targets for the development of the small molecules used to treat a diverse array of medical conditions by influencing their enzymatic activity [42,43]. Considering the essential roles that enzymes play in biochemical pathways and cellular processes, they become prime candidates for drug targeting. In our analysis, we identified a total of 4081 interactions involving 560 enzymes and 1339 drugs, which translates to an average of 3 interactions per drug. However, as shown in Figure 4, there are 21 enzymes with more than 50 drug interactions. These enzymes include carbonic anhydrase isoforms, monoamine oxidase B, cyclooxygenase 1 and 2 (COX1 and 2), and acetylcholinesterase. Except for COX1, all are expressed in CNS tissues, implying that some of the predicted interactions could be repositioned for CNS indications (Figure 5). Of these interactions, 2707 (of 4081) were validated in vitro, indicating a concordance of 66% with experimental data. For this class, we identified a total of 858 interactions out of 2707, with affinities of <100 nM. Among these 858 interactions, there are 176 with affinities ranging from 10 nM to 1 nM, and 57 with affinities <1 nM. Together, these two categories collectively represent 27% of the interactions with affinities less than 100 nM.

### 3.5. Kinases

**Figure 6:**
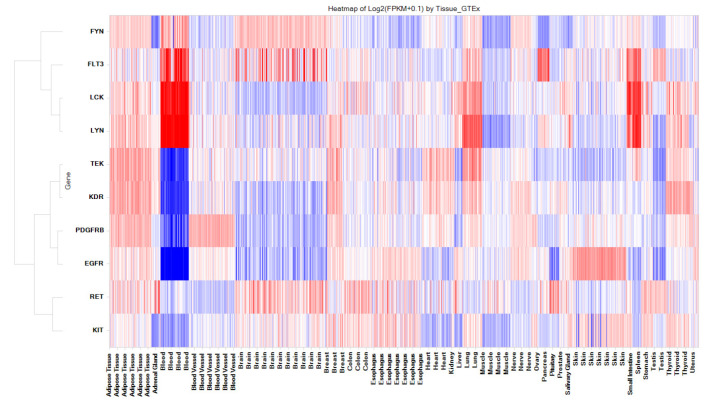
Human Baseline mRNA Expression of Key Off-Target Kinases (10) for 25 Kinase-acting Drugs. The color gradient from red to blue indicates high to low expression levels. FLT, LCK, and LYN are highly expressed in blood, while RET, FYN, and FLT are relatively highly expressed in the brain.

Our analysis revealed a total of 3768 interactions (out of a total of 22,628) involving 427 kinases and 283 drugs (out of 2766), including 53 FDA-approved kinase-targeting drugs. This translates to an average of 13 interactions per drug. Importantly, 3083 of these predicted interactions (out of 3768) have been confirmed in vitro, resulting in an accuracy rate of 81%. The high-affinity interactions have the potential for direct repurposing, while the weaker μM-affinity interactions can be further optimized for better potency through additional synthetic efforts. Among the kinase interactions, we found 1081 (out of 3083) with an affinity of <100 nM. Within this subset, there are 186 interactions with affinities ranging from 10 nM to 1 nM and 49 interactions with affinities less than 1 nM. Taken together, these two categories collectively represent 21% of interactions with affinities less than 100 nM. Furthermore, 17 kinases are potential off-target proteins for over 25 (of 53) drugs, despite the fact that they are primarily targeting different pharmacological kinase and non-kinase targets. The top kinases displaying a sizable number of interactions with FDA-approved drugs include TEK, KDR, PDGFRB, EGFR, FYN, LCK, FLT3, RET, LYN, and KIT. These kinases show expression in various human tissues, including the CNS as shown in the heatmap (Figure 6). Collectively, these results indicate overall that drugs targeting these kinases may have a potential to be repurposed for CNS-related or other indications. Taken together, the confirmation of numerous predicted interactions and the identification of tissue-specific expressions provide valuable insights for potential new therapeutic applications of existing FDA-approved drugs that are intended to target kinases.

### 3.6. Ion Channels

**Figure 7:**
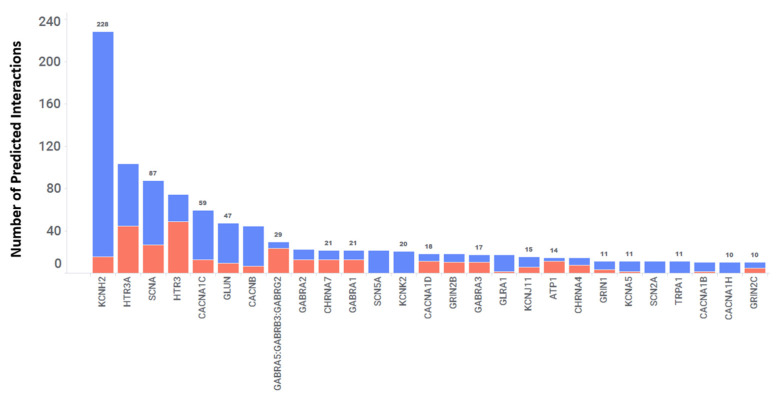
The 27 Ion Channels Predicted to Interact with more than 10 Drug Molecules. The X-axis displays gene names and the Y-axis represents the number of drugs interacting with each gene. For example, 228 drug molecules were predicted to interact with KCNH2 (a potential cardiovascular safety target). The blue segment represents the number of predicted interactions that have been in vitro confirmed. The red segment represents high-scoring predicted interactions that have not been in vitro confirmed to date.

**Figure 8:**
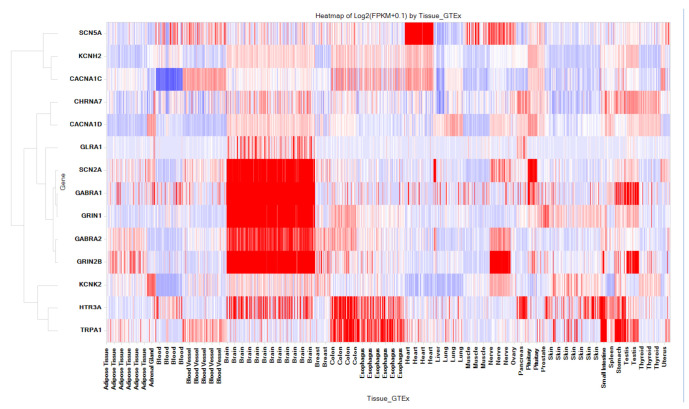
Baseline Human mRNA Expression of key 14 Ion Channels that interacts with more than 10 drugs. The X-axis displays the names of human tissues, and the Y-axis represents gene names. The Y-axis is hierarchically clustered based on gene expression patterns in various tissues.The color gradient from red to blue indicates high to low expression levels.

Ion channels are proteins embedded in cell membranes that form tiny pores allowing specific ions to pass through at fast rates, up to one hundred million per second. These channels are present in nearly all living organisms, ranging from viruses and bacteria to plants and animals, and can be found in various cell types within the human body [44,45]. Approximately 1.5% of an entire genome includes over 400 ion channel genes [46]. This makes ion channels the second largest class of the membrane proteins after G protein-coupled receptors (GPCRs). Out of these ion channels, only a small fraction (~10%) have been targeted for drug discovery efforts. This suggests that a substantial number of ion channels, including those expressed in the CNS, remain unexplored for drug targeting. Hence, we analyzed the predicted interactions in the database between 2766 FDA-approved drugs and ion channels. Our analysis revealed a total of 1303 interactions involving 161 different ion channels and 605 FDA-approved drugs, resulting in an average of 2 interactions per drug. Importantly, 27 out of the 161 ion channels were predicted to interact with more than 10 targets, as shown in Figure 7. These include SCNA, HTR3A, KCNH2, CACNA1C, GLUN, CHRNA7, GABRA2, SCN2A, TRPA1, and others. The heatmap (Figure 8) shows the baseline tissue expression of fourteen ion channels, indicating their expression patterns across different human tissues, including the CNS. Among these predicted interactions, 981 out of 1303 were confirmed with a predicted score of 1, resulting in a 75% agreement with experimental in vitro results. Furthermore, 77 of these interactions have an IC_50_ of <100 nM. Within this subset, there are 29 interactions with affinities ranging from 10 nM to 1 nM and 8 interactions with affinities less than 1 nM. Taken together, these two categories constitute 48% of interactions with affinities < 100 nM. Additionally, there were 322 predicted interactions with a score of 0.6 or higher that have yet to be confirmed through in vitro studies. Interestingly, our analysis revealed that twenty FDA-approved drugs, originally designed to target one or two specific ion channels, were predicted to interact with a total of thirteen ion channels. This suggests the potential for additional interactions and the repurposing of these drugs.

### 3.7. Nuclear Receptors

**Figure 9:**
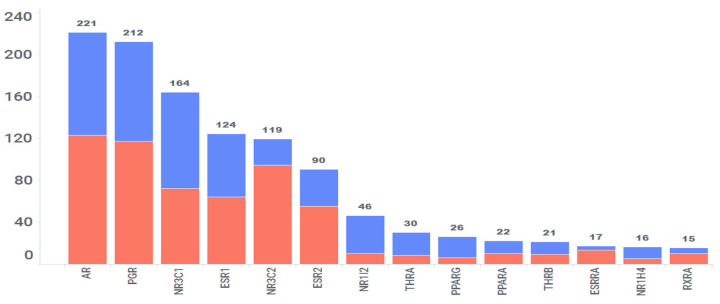
The 14 Key Nuclear Receptors Predicted to Interact with >15 Drug Molecules. The X-axis displays gene names and the Y-axis represents the number of drugs interacting with each gene. The blue segment represents the number of predicted interactions that have been in vitro confirmed. The red segment represents high-scoring predicted interactions that have not been in vitro confirmed to date.

To date, over 300 nuclear receptors (NRs) have been discovered [47], with many identified based on sequence similarity to known receptors but lacking an identified natural ligand, resulting in their classification as “orphan receptors”. In humans, only 48 NRs have been identified so far [48], and several of them fall into the category of orphan receptors. Our computational process predicted interactions with 41 out of these 48 NRs. A total of 1293 interactions were predicted with these 41 NRs, involving 511 drug molecules out of the FDA-approved 2766 drugs studied, translating to an average of 2 interactions per drug. However, 14 NRs were predicted to interact with more than 15 drugs (Figure 9). These NRs include AR, PGR, NR3C1, ESR1, NR3C2, ESR2, PPARα, PPARγ, RXR, and others. Among these predictions, 47% (609) have been confirmed in vitro, and the remaining 53% (684) are considered high-scoring, yet to be confirmed, or refuted. Nevertheless, of the 609 confirmed interactions, 211 interactions have confirmed IC50 values < 100 nM. Within this subset, there are 85 interactions with affinities ranging from 10 nM to 1 nM and 30 interactions with affinities less than 1 nM. Taken together, these two categories constitute 54% of interactions with affinities <100 nM.

Specifically, 221 drugs are predicted to interact with the androgen receptor (AR), and 99 of these interactions have been validated using in vitro studies. AR exhibits a relatively high expression in the liver, female reproductive systems, blood vessels, adipose tissues, testis, pituitary, skin, adrenal glands, and heart, implying the potential repositioning of some predicted AR–drug interactions for diseases related to these tissues. Likewise, 163 drugs were predicted to interact with the glucocorticoid receptor (GR), and 91 of these interactions have been confirmed in vitro. GR is expressed in all key tissues but shows relatively high expression in muscle, blood, adipose tissues, nerves, liver, skin, pituitary, and the brain. Thus, these 163 interactions involving GR can be selectively utilized for potential treatment of the diseases occurring in these tissues.

### 3.8. Cytochromes

The major biotransformation enzyme is Cytochrome (CYP) P450, which comprises more than 1000 isoenzymes. Of these, five (CYP3A4, CYP2D6, CYP2C9, CYP2C19, and CYP1A2) metabolize over 90% of all drugs. Investigating the activity of these CYP isoenzymes (in vitro or in silico) can reveal potential negative side effects caused by drug–drug interactions (DDI) and provide information on the magnitude of the changes due to clinical or preclinical exposure to drug molecules. Our prediction analysis involves predicting interactions with 48 key CYPs, including the five CYPs. Taken together, these two categories collectively represent 26% of interactions with affinities less than 100 nM. After a comprehensive analysis, a total of 2827 interactions were identified involving 48 CYPs and 976 drugs (out of the 2766 FDA-approved drugs explored), translating to an average of 3 interactions per drug. Within these predicted interactions, we identified nine key cytochromes, namely CYP3a4, CYP2d6, CYP2c19, CYP1a2, CYP2c9, CYP2c8, CYP2e1, CYP3a5, and CYP2b6, which displayed interactions with more than 100 drugs. Notably, CYP3a4 displayed 467 interactions, while CYP2d6 displayed 400 interactions. For this class, we identified a total of 116 interactions out of 2227 with affinities <100 nM. Within this subset, there are 21 interactions with affinities ranging from 10 nM to 1 nM and 6 with affinities <1 nM.

**Figure 10:**
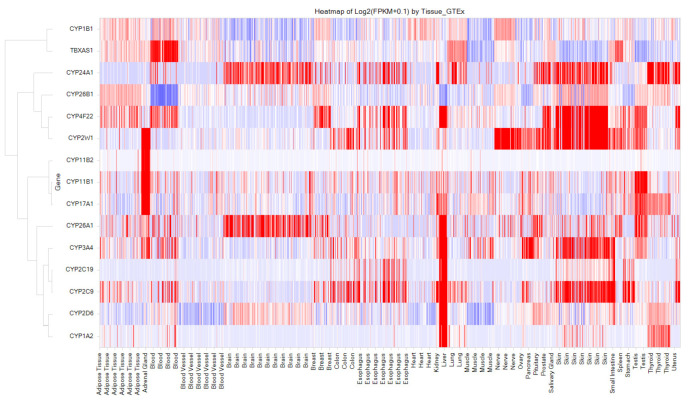
Baseline Human mRNA Expression of key 15 Cytochromes that Interacts with > 100 Drugs. The X-axis displays the names of human tissues and the Y-axis represents gene names. The Y-axis is hierarchically clustered based on gene expression patterns in various tissues. The color gradient from red to blue indicates high to low expression levels.

Additionally, a strikingly high confirmation rate was observed for interactions with the cytochrome target family, with almost 98% (2791 out of 2827) of the predicted interactions having been experimentally confirmed. It should be noted that drugs are not typically developed for CYPs (cytochromes); however, these predicted interactions are valuable in understanding drug metabolism and its potential consequences (both therapeutically and toxicologically). The visual heatmap representation in Figure 10 shows evidence regarding the significant expression of these key cytochromes in the liver, emphasizing their crucial role in drug metabolism. Furthermore, the presence of CYP3e1, CYP2d6, and CYP3c8 in various compartments of the brain indicates the possibility of these cytochromes producing drug metabolites within the CNS, potentially leading to beneficial effects of these later metabolites.

### 3.9. Exploring the Clinically Relevant Off-Target Interactions of 14 Approved Drugs for Repurposing

**Figure 11:**
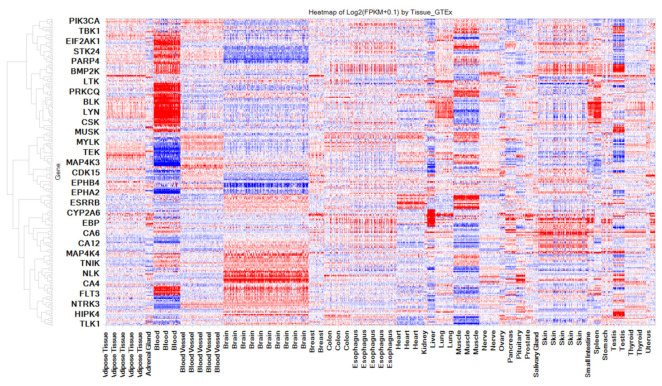
Baseline Human mRNA Expression of Predicted Targets for 14 Selected Drugs. The X-axis displays the names of human tissues and the Y-axis represents gene names. The Y-axis is hierarchically clustered based on gene expression patterns in various tissues. The color gradient from red to blue indicates high to low expression levels.

Nonclinical safety studies of approved drugs often utilize concentrations or doses that generally exceed what may be needed in clinic. Although these high concentrations are valuable in identifying the off-target therapeutic and toxicological effects in nonclinical studies, it is important to have clinically effective concentrations of drugs for meaningful repurposing process [49]. The potential for drugs to be repurposed for different protein targets or therapeutic indications can be informed based on the free plasma concentrations (i.e., C_max_) relevant to clinical use. Consequently, we have compiled human therapeutic concentrations for 14 selected drugs in Table 3, converting them to pC_max_ for easy comparison. We predicted a total of 899 interactions for these 14 drugs. Of these, 859 interactions (with a 95% concordance between prediction and in vitro testing) were in vitro confirmed, translating to an average of 61 confirmed interactions per drug, highlighting the promiscuity of these approved drugs. Interestingly, 7 out of the 14 selected drugs belong to the kinase class, and their unselectivity is expected. Additionally, among the confirmed interactions, 113 showed high affinity with an IC_50_ < 100 nM. The remaining 658 interactions ranged from 100 nM to 1 μM. Furthermore, within this subset, we found 30 interactions with affinities ranging from 10 nM to 1 nM and 9 interactions with affinities less than 1 nM. Taken together, these two categories collectively represent 34% of interactions with affinities less than 100 nM. Next, we compared these values with predicted off-targets and the pIC_50_, which represents the concentration needed to inhibit 50% of the target activity. If the pIC_50_ is lower than the pCmax, it indicates that the drug’s impact on the predicted off-target could have notable consequences. We have included in Table 3 only off-targets with pIC_50_ values below 6, indicating μM IC_50_ values or less. The heatmap (Figure 11) shows the baseline tissue expression of the key off-targets of 14 selected compounds, displaying their expression patterns across different human tissues, including the CNS. For the four selected compounds, the predicted off-target associations with various diseases are provided in Supplementary Tables S2–S5. For example, the predicted off-target effect of GAK for Bosutinib is associated with Parkinson’s disease, Hepatitis C, and osteosarcoma of the bone (see Supplementary Table S2). The diseases associated with other predicted targets of Bosutinib are also provided.

## 4. Discussion

Traditional drug discovery focuses on a one-drug to one-gene or one-protein tactical approach, which fails to consider the polypharmacological properties of small molecules. This drug-hunting method is both time-consuming and expensive. On the other hand, drug repurposing offers a promising alternative by identifying existing drugs that could serve new therapeutic purposes, making the discovery process more rapid and cost-effective [19,50]. One significant advantage of drug repurposing is the potential for the quicker introduction of new therapeutics to patients in need [21,51], by avoiding the lengthy new chemical lead candidate identification and toxicological battery testing that are traditionally required. Moreover, since these drugs have already been evaluated in toxicity studies in animal models, the risk of the identification of new, adverse, and/or non-reversible findings that may translate to humans is significantly minimized.

Pharmaceutical companies are now increasingly focusing on drug repurposing [52,53,54,55]. However, relying solely on serendipitous discoveries, like sildenafil transitioning from an anti-angina drug to an erectile dysfunction treatment [56], is not a reliable and sustainable approach. Instead, innovative methods have been developed to combine experimental and targeted computational techniques, such as in silico chemical and large data analyses, followed by in vitro validations before testing in disease-relevant animal models and subsequently in humans [17,57,58]. Our approach involves multiple chemical similarity and AI/ML techniques to identify all potential interactions for small molecules relative to the human genome. Notably, we leverage tissue-specific transcriptomics data from various species, including humans, cynomolgus monkeys, rats, mice, and dogs, to understand the predicted interactions in tissues for pharmacologically relevant drug repurposing. The results from our approach have been promising, as they align well with in vitro binding activities for many predicted interactions, giving us confidence in our method.

Our preliminary analysis indicates that neither logP, TPSA, nor other computed physicochemical properties, influence drug promiscuity (Table 1), i.e., the predicted number of off-target interactions. For example, romidepsin, an anticancer compound, is predicted to interact with 27 targets, with logP and TPSA values of 2.3 and 153 A^o2^, respectively. Propafenone, an antiarrhythmic drug, is predicted to interact with 28 targets, with logP and TPSA values of 3 and 63 A^o2^, respectively. This implies that compounds are often developed based on a benefit-to-risk criterion, independent of certain chemical properties. Furthermore, our current study focuses on leveraging predicted off-target interactions exclusively within the context of repurposing. Nevertheless, we plan to explore the potential toxicity associated with these interactions in a follow-up study, providing a comprehensive understanding of the benefit-to-risk ratio in repurposing drugs for non-oncology indications.

In the following sections, we discuss the importance and feasibility of the off-target interactions we have identified for potential drug repurposing, with a specific focus on kinases and GPCRs. We have also collected any relevant clinical or nonclinical evidence to substantiate these potentially repurposable drugs. Furthermore, we analyzed the feasibility of using drugs previously approved as a human therapeutic in animals, evaluated the significance of the predicted interactions during the due diligence process, and explored the potential impact of predicted interactions on compound life cycle management. Lastly, we provide select limitations and challenges associated with the drug repurposing process.

### 4.1. Predicted Interactions and Repurposing Opportunities

Remdesivir is a broad-spectrum antiviral medication that has recently been identified as a potential treatment for COVID-19. Our computational approach identified DNA polymerase as a potential target with a computed score of 0.69, implying high confidence. Additionally, we predicted four potential metabolites, which were predicted to bind with the Adenosine receptor 2 (A2AR), P2RY12, and GPR139. A2AR is involved in protecting the lungs from high neutrophil concentrations and increased vascular permeability. The latter can lead to the leakage of exudate, preventing proper gas exchange. Thus, A2AR agonists are useful in treating asthma and chronic obstructive pulmonary disease by blocking the flooding of the bronchioalveolar space with white cells and suppressing the activation of inflammatory cells, thereby reducing the extent of immunosuppression. A recent study suggested that the engagement of this hypoxia-triggered pathway by drugs such as adenosine ligands of A2A and A2B adenosine receptors may prevent the exacerbation of inflammatory lung damage and improve outcomes in oxygen-ventilated COVID-19 patients [59]. Taken together, our predicted interaction between remdesivir metabolite and A2AR agrees with this published study, and we suggest that this interaction may contribute to the observed reduction in COVID-19 viral infection using remdesivir. Additionally, our approach predicted that telbivudine (an antiviral drug) and fenoldopam are potent modulators of A2A, and we suggest the potential utility of these A2AR drugs for COVID-19 as well.

Benserazide, primarily used to treat Parkinson’s disease (PD), has been identified as a potential treatment for triple-negative breast cancer through computational methods [60,61]. This discovery has been validated in nonclinical studies, and ongoing clinical trials aim to further explore its effectiveness in treating this specific type of cancer. Our computational framework predicted five targets (DDC, SAE, ALP1, ESR2, Mex-5, and Tat) for Benserazide, and these predictions have been confirmed through in vitro experiments. Of particular interest, DDC, TAT, and ESR2 display high expression in CNS tissues, specifically in the hippocampus and cerebellum of the brain, and the spinal cord. This suggests that the off-target interactions of Benserazide may play a significant role in treating various CNS-related conditions. Recent studies support these findings, demonstrating that Benserazide shows promise in improving motor function, reducing neuroinflammation, and enhancing cognitive function in animal models of PD and Alzheimer’s disease (AD). As noted above, we also identified SAE1 as a potential off-target for Bensazeride; it plays a critical role in regulating triple-negative breast cancer (TNBC). Wang et al. [62] found significantly elevated mRNA and protein levels of SAE1 in TNBC tissues compared to adjacent normal tissues. SAE1 protein expression significantly relates to overall and disease-free survival. This aligns with our prediction, supporting the exploration of Bensazeride for the treatment of TNBC based on predicted and in vitro-confirmed SAE1 interaction.

Several endocrine drugs have been repurposed for CNS indications. Pioglitazone, an FDA-approved drug used in the treatment of type 2 diabetes, has shown potential in nonclinical studies for its neuroprotective effects and effectiveness in treating AD [63]. This drug has been found to reduce inflammation in the brain, improve cognitive function, and reduce the accumulation of amyloid beta plaques, which are characteristic of AD. We have identified 13 interactions associated with pioglitazone, involving the following enzymes and proteins: CYP2C19, CYP2C8, CYP2C9, CYP2D6, CYP3A4, MAOB, CA2, PPARγ, PPARα, RXRA, PPARγ, SLC10A1, ABCB11, and CISD1, which have been confirmed in vitro. Interestingly, these targets are expressed in CNS tissues; MAOB, ABCB11, PPARα, and CA2 show relatively higher expression levels in the CNS relative to peripheral organs. These findings provide new insights on interactions that may potentially be responsible for the observed favorable CNS effects of pioglitazone and its potential repurposing as an endocrinology-acting compound for potential CNS benefits due to polypharmacology.

Metformin is an oral medication commonly used to treat type 2 diabetes. Recent nonclinical studies have shown that metformin also has neuroprotective effects, leading to its investigation as a potential treatment for AD and other neurological disorders [64,65]. Our computational analysis predicted four potential targets for metformin: GPD2, BACE1PPP2CA, USP7, and MID1:ALPHA4: PP2A. These predicted interactions have been validated and confirmed using in vitro assays. Additionally, all four targets, which were both predicted and confirmed in vitro, are expressed in CNS tissues. Of these, BACE1 shows significantly high expression across all CNS compartments, suggesting a strong link between metformin’s interactions and in vivo CNS effects. Furthermore, USP7 is known to be involved in the replication of the SARS-CoV-2 virus, which is highly relevant to the context of COVID-19. Our predicted interaction between metformin and USP7 suggests that metformin may potentially inhibit virus replication by targeting USP7. However, further in vivo studies are necessary to confirm this binding hypothesis and determine the potential clinical applications of metformin as an oral anti-COVID drug. Notably, early outpatient COVID-19 treatment with metformin decreased the subsequent risk of long COVID by 41.3% during a 10-month follow-up. which was consistent with the 42.3% reduction in health care utilization for severe COVID-19 in the first 14 days of the trial [66].

### 4.2. Repurposing GPCR and Kinases

GPCRs and kinases are two distinct classes of proteins that play crucial roles in cell signaling pathways. While they typically interact with different classes of ligands, there are some examples of drugs that can bind to both GPCRs and kinases.

Our drug repurposing process has the potential to be highly beneficial in drug repurposing efforts for GPCR. These receptors play a vital role in cellular responses to hormones and neurotransmitters and are the largest group of therapeutic targets for various diseases. Although most approved drugs target GPCRs, such as those for major disease indications like diabetes, oncology, obesity, AD, and other CNS disorders, there is still much untapped therapeutic potential.

Imatinib, originally designed as a kinase inhibitor to target the BCR-ABL fusion protein in chronic myeloid leukemia, was later found to also activate the serotonin 4 (5-HT4) receptor, a GPCR associated with gastrointestinal function. This dual action of imatinib is believed to contribute to its effectiveness in addressing gastrointestinal stromal tumors (GISTs), as these tumors express elevated levels of the 5-HT4 receptor. Interestingly, our analysis did not identify 5-HT4 as an unintended target; instead, we predicted interactions with two other GPCRs: HRH2 and HTR2A. Furthermore, we predicted 68 additional targets, all of which were confirmed in vitro. These targets span diverse target classes: (1) 8 cytochromes, (2) 15 enzymes, (3) 5 transporters, (4) 4 unclassified proteins, and (5) 36 distinct kinases. These findings indicate the inherent promiscuity embedded in this compound. Therefore, we suggest that the observed efficacy of imatinib in modulating gastrointestinal effects could be attributed not only to its potential interaction with 5-HT4 but also to its engagement with various off-targets, especially kinases and predicted GPCRs expressed in gastrointestinal tissues.

Sorafenib inhibits several kinases involved in cell signaling pathways, including Raf kinase and VEGFR (vascular endothelial growth factor receptor). It is used to treat advanced kidney and liver cancers. Sorafenib has also been shown to bind to the adenosine A3 receptor, a GPCR involved in immune regulation, and this interaction may contribute to its anti-inflammatory effects. Our computational framework predicted 121 targets for this compound and of these 120 are confirmed in vitro. These include A3, VEGFR, and Raf kinases as well (Supplementary Table S1).

Ibrutinib, a BTK inhibitor developed to treat chronic lymphocytic leukemia and small lymphocytic lymphoma, has shown promise in treating aggressive glioblastoma by interacting with BMX. Additionally, ibrutinib is emerging as a potential drug to treat certain autoimmune conditions. Ibrutinib’s action on B-cell signaling pathways, implicated in autoimmune problems like rheumatoid arthritis and systemic lupus erythematosus, is notable. Recent studies indicate that it can modify cocaine-induced startle responses and reduce cocaine-triggered seizures, with significant participant numbers (61 to 142 per group, 51% females). These findings highlight Ibrutinib’s overall potential to address cocaine-related neurotoxicity [67]. Our analysis revealed 34 off-targets, 29 of which are kinases, including key players like BTK, BMX, and BLK. These are expressed in both peripheral and central nervous system tissues, suggesting their applicability in treating CNS (including seizure) and non-CNS disorders (e.g., neuroimmunology and neuro-oncology).

Niclosamide is an anthelmintic medication used to treat tapeworm infestations, including diphyllobothriasis, hymenolepiasis, and taeniasis [68]. The mechanism of action of niclosamide has been demonstrated to block glucose uptake, thus acting as an uncoupling agent for energy-generating oxidative phosphorylation in intestinal worms, effectively starving the worms of ATP. Our computational framework predicted that this drug interacts with 16 targets, including 4 CYPs, 5 enzymes, 4 G protein-coupled receptors (GPCRs), 1 ion channel, 1 nuclear receptor, and 1 other protein family. Importantly, the predicted interaction with metabotropic glutamate receptor 5 (mGlu5-a GPCR), which has been confirmed in vitro, is particularly intriguing within the context of oral COVID therapy. Recently, Singh et al. [69] suggested the potential use of niclosamide for COVID treatment. However, the specific mechanism underlying this use was not indicated in their work. We propose that the interaction between niclosamide and mGlu5 may be a key factor in considering this drug for oral COVID therapy.

Furthermore, there are drugs that originally target ion channels, which have been repurposed to other indications by targeting GPCR targets.

Amlodipine, for example, is a calcium channel blocker used to treat hypertension and angina. However, it has been found to also act on GPCRs. Amlodipine is being investigated as a potential treatment for PD through its interaction with D2, which is predicted via computational process (Supplementary Table S1). Amlodipine is predicted to engage with a variety of targets (27 targets, 26 confirmed) within the CNS and peripheral tissues, including alpha adrenergic receptors (2A and 2C), HTR6, the sodium-dependent dopamine transporter, calcium channels (CACNA1D, CACNB, CACNA1C, and CACNA1B), and muscarinic acetylcholine receptors in the CNS. For example, the dopamine transporter facilitates the movement of extracellular dopamine into the intracellular space, contributing to the regulation of dopamine neurotransmission. Changes in the density of this transporter have been associated with PD. Thus, dopamine turnover is elevated in the early stages of symptomatic PD and in individuals with monogenic mutations that lead to parkinsonism. Furthermore, the predicted involvement of muscarinic acetylcholine receptors is also significant in the context of PD [61]. Additionally, the presence of 5-HTR type 6 (5-HTR6) on postsynaptic 5-HT neurons in various brain regions, such as the basal ganglia, cortex, and limbic system, as well as on cholinergic and GABAergic neurons in the striatum, highlights its role. Preclinical studies involving 5-HTR6 suggest its contribution to regulating learning and memory, possibly through the stimulation of glutamatergic and cholinergic transmission, providing potential implications for PD and other CNS diseases [70]. These CNS-relevant predicted targets collectively provide a potential rationale for investigating the use of this drug in treating CNS disorders.

Pregabalin is another example. It is an anticonvulsant medication used to treat neuropathic pain and epilepsy, and it modulates the α2δ subunit of voltage-gated calcium channels. Pregabalin has also been shown to interact with the CNS, expressing GPCR. This drug is currently being studied as a potential treatment for anxiety disorders via its interaction with the CNS, expressing GPCR targets. We predict that this drug interacts with four targets: GABABR (although not yet confirmed in vitro), as well as three ion channels (CACNA2D2, CACNA2D4, and CACNA2D1—all of which have been confirmed and are intended targets). Several reports have linked GABABR to conditions like neuropathic pain and epilepsy [71]. Therefore, we suggest that the predicted interaction with GABABR (a GPCR target) could potentially work synergistically with the intended ion channels, leading to a significant improvement in effectiveness for treating anxiety, epilepsy, and neuropathic pain.

Taken together, these studies and examples provide evidence for the potential of repurposing existing drugs from one indication to another, which can be accomplished effectively with the help of nonclinical and clinical studies. This approach can lead to significant reductions in both the cost and time required for drug discovery. Ultimately, such a strategy can help accelerate the development of new treatments for various diseases and conditions.

The functional activation of 5HT2B and PPARγ, as well as the inhibition of hERG and cKIT tyrosine kinase, can lead to safety concerns. For instance, 5HT2B agonists have been associated with the development of clinical valvular heart disease [24]. Activation of PPARγ has been linked to the occurrence of cardiac and hepatobiliary disorders [72]. Additionally, the blocking of the hERG channel has been implicated in QT prolongation, and inhibiting cKIT kinase has been connected to conditions such as anemia, neutropenia, and thrombocytopenia [73].

Our analysis revealed that 277, 26, 228, and 29 drugs interact with 5HT2B, PPARγ, the hERG channel, and cKIT tyrosine kinase, respectively (Supplementary Table S1). It is important to note that our computational prediction is specifically designed to determine whether a compound binds to a particular target or not. It does not predict the functional effects of these interactions (agonist, antagonist, or partial agonist). Consequently, we suggest that when using the data described in Supplementary Table S1 for interrogating potential drug repurposing, further investigations to reveal the significance of the evidence should include functional (in vitro) assays as well as efficacy (in vivo) studies, where needed/feasible to reveal the actual effects of these predicted interactions with target(s). If the functional effects are agonistic for 5HT2B and PPARγ for any drug and potent inhibitors of hERG or cKIT, we suggest deprioritizing them for repurposing to minimize resources on efforts that may result in potential development failure(s).

### 4.3. Repurposing Human Drugs for Animals

When repurposing drugs previously approved as a human therapeutic for use in animals, there are several considerations that need to be considered to ensure safety and efficacy. One important consideration is the pharmacokinetics (PK) and pharmacodynamics (PD) of the drug in the target animal species, which generally are different than in humans. Additionally, the dosage and potential adverse effects of the drug need to be carefully evaluated to ensure that the animal is receiving the appropriate treatment. Nevertheless, the advantages of repurposing human drugs for animals include the potential for faster drug development, leading to earlier marketing, as existing extensive knowledge about the safety and efficacy of the drug in humans and earlier GLP toxicology data can be effectively leveraged. This can also lead to cost savings and a reduced need for further animal testing in the drug repurposing process. For example, prednisone is a corticosteroid commonly used in humans to treat inflammation and immune-related disorders [74]. It has also been used in dogs to treat various conditions such as allergies, inflammatory diseases, and cancer [75]. Our computational process predicted that prednisone interacts with several targets, including Tyrosine aminotransferase, Platelet-derived growth factor receptor beta, Vascular endothelial growth factor receptor 2, Glucocorticoid receptor, and Sex hormone-binding globulin. Among these targets, Angiopoietin-1 receptor, Mineralocorticoid receptor, and Sex hormone-binding globulin have not been confirmed in vitro. However, others such as PDGFR, VEGFR2, GR, and TEK are in vitro-confirmed and are highly validated oncology and immunology targets, offering a reason to use prednisone to treat cancer and immune-related indications in dog.

#### 4.3.1. The Drug Repurposing Role in the Due Diligence Process

The drug repurposing process is an important due diligence step when considering making deals or analyzing external companies compounds [76]. This process involves evaluating the safety and efficacy of a drug candidate in a new indication or target population. The first step in drug repurposing during the due diligence process is to identify potential drug candidates based on a review of their existing clinical data, polypharmacological activity data, or other factors. Next, pilot nonclinical, and/or clinical testing is conducted to evaluate the drug’s safety and efficacy in the new indication or population. This may involve conducting short animal studies, as well as clinical trials in humans. Additionally, regulatory considerations need to be considered, including the intellectual property in a new indication, regulatory approval pathways, and most importantly the market potential. We suggest the development of an integrated framework that includes predicted and confirmed protein interactions, target expression, target biology, as well as in vitro, ex vivo, pathology, preclinical, and clinical data, on the compound of interest. This comprehensive approach will aid in making well-informed decisions during the due diligence process. For instance, consider the collaboration between Merck and Ridgeback Biotherapeutics. Merck entered a partnership with Ridgeback Biotherapeutics to develop a potential treatment for Ebola [77]. The drug, EIDD-2801, was initially designed for influenza treatment and has been repurposed for Ebola.

#### 4.3.2. Drug Repurposing in Compound Life Cycle Management

Drug life cycle management through repurposing involves identifying new therapeutic uses for approved drug molecules, extending their life cycle and value by leveraging established safety profiles, institutional knowledge and known and new predicted mechanisms of action [52]. Compared to developing new drugs, repurposing is a more efficient and cost-effective alternative, bypassing nonclinical and clinical development stages and reducing costs and time to market. For example, new indication for internal drug molecules can extend their patent life, ensuring market exclusivity and protection against generic competition on the composition of matter (Reference: Supplementary Table S1 for drug–target interactions). Additionally, new indications on the drugs have the advantage of established, internally generated, nonclinical and clinical safety data, enabling faster regulatory approvals, and potentially minimizing risks. Predicted new drug interactions may also address unmet medical needs with limited treatment options. Including repurposed drugs enhances a company’s portfolio, showing their ability to identify opportunities, maximize assets, and adapt to market demands. Overall, drug repurposing optimizes life cycle management, identifies new therapeutic uses, extends market life, and benefits both pharmaceutical companies and patients.

#### 4.3.3. Limitations to Drug Repurposing

As discussed, the concept of drug repurposing offers a promising approach in the field of pharmaceutical development. However, it is important to acknowledge the inherent limitations that come with this strategy. Repurposing involves repackaging existing drugs for new therapeutic applications, with a goal to save time and resources. However, the seamless optimization of a drug for a new purpose is not guaranteed. This is due to the difference between the binding pocket of the drug’s primary intended target and the binding site anatomy of the new target being considered for repurposing. These differences can impede the previously established SAR tailored for the original target’s binding site, resulting in potential challenges to achieve the desired therapeutic effects of the new target. Consequently, repurposing might yield only temporary solutions, necessitating synthetic modifications to enhance compatibility. Medicinal chemists play a major role in the repurposing process. They can exploit the starting lead provided by existing drugs, designing more potent molecules for new targets. This will involve exploring analogs, which have been synthesized but show only weaker affinity for the original target, which can be repurposed for the new target. Furthermore, the predicted and in vitro-confirmed binding data alone are insufficient to assess repurposing opportunities. Various factors, such as efficacious systemic exposure levels, the volume of distribution, tissue expression, and off-target residence time, must be taken into consideration when assessing the potential for repurposing based on predicted off-targets [78].

For example, in this work, we studied Afatinib, an approved kinase-targeting drug commonly used to treat non-small cell lung cancer [47]. At the recommended daily dosage of 40 mg, Afatinib achieves a maximum concentration in the bloodstream (Cmax) of 0.052 µM, corresponding to a pCmaxIC50 of 7.2. Using our computational approach, we predicted eleven off-targets that displayed a measured pIC_50_ > 6.0 (ranging from µM to nM). Given that the therapeutic pCmax IC50 for Afatinib is 7.2 at the clinical dose, it is plausible that these predicted and confirmed off-target interactions could offer additional therapeutic benefits for various tissue-specific diseases. Moreover, transcriptomic analysis further revealed that the predicted off-target kinases are expressed in tissues relevant to other diseases, particularly in CNS tissues (Supplemental Figure S1).

For instance, if we have additional information such as the drug residence time within the binding pocket of the predicted target(s) for repurposing, it may help us understand whether prolonged efficacy is likely or not. Several examples confirm the role of residence time in prolonged target-mediated in vivo outcomes [78]. The need to integrate these data with off-target interaction information represents the future of drug repurposing prioritization based on the mechanism of interest. Furthermore, this integrated information offers us the opportunity to transform data into useful knowledge for successful drug repurposing. We suggest that these additional features enable the formulation of more robust repurposing hypotheses related to mechanisms of action.

Furthermore, the ligand-centric (chemical similarity-based) and AI/ML methods within the OTSA framework inherently have bias based on the chemical data available in published sources such as patents and publications. This bias significantly restricts their predictive capability for novel chemical compounds that are not represented in the training set. Another drawback of ligand-centric similarity-based approaches is the potential for false positive predictions arising from structurally similar but inactive compounds. To tackle this issue, we have implemented orthogonal methods, each of which captures complementary and supplementary pharmacophoric information to align with in vitro activities. Additionally, we continuously expand the training set to enhance both the diversity of chemical data and biological activity.

#### 4.3.4. Challenges in Drug Repurposing

Limited data sharing: Many pharmaceutical companies are reluctant to share their repositories of unused or unsuccessful drugs. This scarcity of available options restricts the search for viable repurposing candidates.

Physicochemical properties: some drugs possess unfavorable physicochemical properties, such as poor solubility or lack of specificity, which hinder their suitability for new applications like in the CNS.

Concerns about adverse effects: repurposing can introduce an increased risk of adverse effects when drugs are used in different doses or among diverse patient populations.

Complex modifications: Repurposing often involves modifying the drug’s chemical structure to suit the new purpose, as indicated. Additionally, adapting a drug designed for adults for use in pediatrics requires careful adjustments.

Legal and regulatory complexity: the intricate landscape of legal, regulatory, and patent-related considerations further complicates the drug repurposing process.

#### 4.3.5. Failed Attempts

Several examples illustrate the challenges of drug repurposing. For example, Hydroxychloroquine, an antimalarial drug, gained significant attention as a potential treatment for COVID-19 based on some preliminary positive findings [79]. However, subsequent well-designed clinical trials did not show a significant benefit of hydroxychloroquine in treating COVID-19. Furthermore, some studies even highlighted potential risks and side effects associated with its use. In yet another example, celecoxib, a nonsteroidal anti-inflammatory drug (NSAID) used for pain relief, was investigated for its potential in treating AD due to its anti-inflammatory properties. However, clinical trials did not demonstrate significant cognitive benefits in AD patients treated with celecoxib, leading to the conclusion that it is not an effective therapy for this neurodegenerative disorder.

To increase the probability of success, we recommend adopting an integrated approach. This approach entails combining in silico predicted off-targets with subsequent in vitro validation, incorporating tissue expression data from various species, including humans, rodents, and non-rodents. It also involves analyzing existing evidence in the literature, integrating available in vivo toxicology and pathology information generated for the drug, and considering gene knockout phenotype data when they are available. This comprehensive framework has the potential to improve the regulatory and technical success rate of drug repurposing activities, making the process more efficient and effective.

## 5. Conclusions

In summary, drug repurposing represents a promising and efficient strategy for the pharmaceutical industry to address the challenges of high attrition rates and substantial investments in drug discovery. By identifying new therapeutic uses for existing FDA-approved drugs, drug repurposing offers a cost-effective and time-efficient alternative to traditional drug development. In this study, we developed a computational framework that utilizes AI/ML-based target prediction methods and transcriptomics to predict potential interactions and tissue expression information for 2766 FDA-approved drugs. Applying this framework has led to the identification of numerous off-target interactions, presenting new repurposing opportunities. Encouragingly, a considerable number of these predictions were experimentally confirmed in vitro, with a confirmation rate of 63%. These findings represent a valuable resource for the pharmaceutical industry (Supplementary Table S1) and open new avenues for drug repurposing, enabling the treatment of various diseases and conditions through the discovery of unintended targets. Our investigation into unintended targets has suggested new therapeutic possibilities, which may lead to a greater success rate for drug development and expedited patient benefit. However, it is important to acknowledge the limitations of our strategy. For instance, if a drug was initially developed for a different purpose than its proposed new use, it may not be optimized for the new application due to differences in binding site anatomy and features. Additionally, repurposed drugs can only serve as a starting point from which additional drug properties can be synthetically tailored to be compatible with the new target’s binding pocket. In some cases, additional synthetic modifications to approved drugs and in vitro/in vivo follow-up studies may be necessary for effective engagement with unintended targets.

## Figures and Tables

**Table 1:** Computed physicochemical properties of 2766 FDA-approved drugs.

Property	Mean	Median	Minimum	Maximum
MW	348.4	325.4	60.0	994.1
logP	2.6	2.8	−9.1	16.7
logS	−3.8	−3.7	−19.6	6.1
Caco2	981.0	383.5	0.0	9906.0
MDCK	1033.3	315.0	0.0	10,000.0
Number of Metabolites	3.8	4.0	0.0	17.0
TPSA	80.5	72.5	0.0	446.5
HBD	1.7	1.0	0.0	19.0
HBA	6.0	5.25	0.0	33.2
Amides	0.15	0.0	0.0	11.0
Number of rotatable bonds	5.7	5.0	0.0	37.0

**Table 2:** Predicted interactions of 2766 FDA-approved drugs with therapeutic target classes.

Target Class	Total Predicted Interactions	Predicted (Unconfirmed)	Predicted and In Vitro-Confirmed	% Confirmed Predictions
GPCR	10,650	5708	4942	46
Enzymes	4081	1374	2707	66
Kinase	3768	688	3080	81
Nuclear Receptor	1293	684	609	47
Other Families	605	197	408	67
Transporter	1788	651	1137	63
Unclassified	1057	429	628	59
Cytochrome	2827	36	2791	98
Ion Channel	1303	322	981	75

**Table 3:** Summary of predicted interactions for selected approved drugs.

Generic Name	Intended Pharmacological Target (s)	C_max_ (μM)	PC_max_	Number of Predicted Off-Targets with Measured pIC50 > 6.0	Key Predicted Off-Target Interactions
Afatinib	EGFR, HER2, HER4	0.0520	7.2800	11	EGFR, ERBB4, ERBB2, GAK, BLK, IRAK1, EPHA6, HIPK4, PHKG2, LCK, ABL1
Bosutinib	BCR-Abl, Src	0.3770	6.4200	>50	ABL1, MAP4K5, ERBB3, LCK, ABL, GAK, ABL2, FRK, STK35, SRC
Celecoxib	COX-2	4.600	5.3400	19	INSR, CA9, CA12, CA, Ca15, MT-CO2, ACA7, CA2, CA5B, CA6, CA13, NCE103, PTGS2, CA1, CA4, PTGES, CA14, A6YCJ1, CA5A, MAPK14
Ceritinib	ALK	1.2100	5.9200	17	NUAK1, MAP4K4, ACVR1, AXL, PAK4, TYK2, PHKG1, PTK2B, DAPK3, DAPK1, HIPK1, MUSK, TAOK1, RPS6KA4, SRPK3, HIPK4, FRK
Erlotinib	EGFR	3.1500	5.5000	25	GAK, MAP3K19, EGFR, SLK, STK10, MAP2K5, RIPK2, LCK, ABL1, BLK, LYN, SLCO2B1, TNNI3K, TNK1, CIT, MKNK1, ULK3, JAK3, DDR1, ERBB4, TIE1, EPHA6, ERBB2, PIP4K2C, ABCB11
Finasteride	5-Alpha Reductase	0.1240	6.9100	4	SRD5A2, STRD5, SRD5A1, NLRP1
Gefitinib	EGFR	0.3560	6.4500	21	GAK, EGFR, IRAK1, ERBB4, MAP3K19, RIPK2, MKNK1, SIK2, TUBA1A, MKNK2, SBK1, MAP2K5, HIPK4, IRAK4, STK10, CHEK2, ERBB3, ERBB2, LYN, LCK, KDR/VEGFR
Hydroxychloroquine	Cathepsin L	0.3500	6.4600	8	MPO, CHRM2, ADRA1D, TLR9, TLR8, TLR7, CRYAB, TLR4
Imiquimod	TLR7R	0.0056	8.2500	5	HRH2, ADRA1D, ADORA2A, HCAR1, TLR
Lapatinib	ERBB2, EGFR	4.1800	5.3800	11	EGFR, TUBA1A, ERBB2, PIK3CA, NRAS, BRA, ERBB4, PIK3C2B, KRAS, PI4KB, MAP2K5
Olaparib	PARP	13.1000	4.8800	9	PARP11, PARP2, PAR16, PARP10, PARP1, PARP3, PARP4, RAD51, TNKS
Sirolimus	Mammalian target of rapamycin (mTOR)	0.0160	7.7800	12	FKBP1B, FKBP1A, FKBP5, PIK3, ABCB1, MTOR, TEK, NR1I2, EIF4E, PSM, SLCO1B1, ABCB11
Tamoxifen Citrate	Estrogen Receptor	0.1080	6.9700	21	EBPL, SIGMAR1, EPHX2, ESRRG, EBP, ESR2, ESR1, ESRRA, HTR2C, DRD3, KCNH2, TBXAS1, PER1, HTR6, ADRA2A, FYN, CHRM3, CHRM1, ERG, SLC6A2, ERG2
Teniposide	Topoisomerase II	23.1000	4.6400	9	NCOA3, TOP2, NCOA1, AR, ESR1, ESR2, PGR, THRA, THRB

## Data Availability

Where not included in the main body or as Supplementary Materials, the data that support the findings of this article may be made available on request from the corresponding author (Mohan Rao). Select data are not publicly available due to restrictions, such as containing information that could compromise select intellectual property.

## Supplementary Materials

The following supporting information can be downloaded at: https://www.mdpi.com/article/10.3390/toxics11100875/s1, Figure S1: Human Baseline mRNA Expression of Predicted Off-Targets of Afatinib. Different regions of various tissues are displayed on the X-axis. For example, HIPK4, EPH6, and ERBB4 show high expression levels in distinct brain regions. Table S1: Compilation of 27,371 predicted interactions for 2766 drug molecules. This table contains all metadata, including drug names, target names, experimental pIC50 values (if known), predicted pI50 values (for all interactions), predicted scores (only for interactions with a score of 0.6 or higher), and the number of methods that predicted each specific interaction. Table S2: Potential therapeutic indications for Bosutinib’s off-targets. Each tab in this Excel sheet contains disease-gene associations for targets, including gene-disease association scores. A higher score indicates greater confidence. Table S3: Potential therapeutic indications for Celecoxib’s off-targets. Each tab in this Excel sheet contains disease-gene associations for targets, including gene-disease association scores. A higher score indicates greater confidence. Table S4: Potential therapeutic indications for Certinib’s off-targets. Each tab in this Excel sheet contains disease-gene associations for targets, including gene-disease association scores. A higher score indicates greater confidence. Table S5: Potential therapeutic indications for Erlotinib’s off-targets. Each tab in this Excel sheet contains disease-gene associations for targets, including gene-disease association scores. A higher score indicates greater confidence.
